# Endothelial cells influence the sodium nitroprusside mediated inhibition of platelet aggregation by an as yet unkown pathway

**DOI:** 10.1186/1477-9560-10-6

**Published:** 2012-05-07

**Authors:** Hans-Georg Topf, Manfred Rauh, Wolfgang Rascher, Jörg Dötsch, Jens M Klinge

**Affiliations:** 1 Klinik für Kinder und Jugendliche, University of Erlangen-Nuremberg, Loschgestr 15, 91054, Erlangen, Germany

**Keywords:** Nitric oxide (NO), Platelet aggregation, Sodium-nitro-prusside (SNP), Cyanide, Endothelial cells

## Abstract

The clinical use of Sodium nitroprusside (SNP) may be associated with an alteration of platelet function. The main focus of this study was the effect of SNP on platelet aggregation in the absence or presence of endothelial cells.

Methods: Platelets were incubated with different concentrations of SNP with and without endothelial cells. Platelet aggregation was induced by ADP.

Results: Platelet aggregation was significantly inhibited by all concentrations of SNP. Endothelial cells significantly increased this inhibitory effect of SNP. Time course studies showed an inverse correlation of incubation time to platelet aggregation inhibition in the absence of endothelial cells, and a direct correlation in the presence of endothelial cells. Blocking platelet and endothelial cell guanylate cyclase with 1 H-(1,2,4)-oxadiazolo(4,3-a) quinoxalin-1-one (ODQ), or pretreatment of the endothelial cells with cyclooxygenase – inhibitors, had no influence on the increased inhibitory effect of the endothelial cells. Cyanide reversed the inhibitory effect of SNP completely.

Conclusion: Endothelial cells play an important role in the SNP mediated inhibition of platelet aggregation. The effect is reversible only by cyanide, not by blocking classical NO signal transduction.

## Introduction

The administration of Sodium nitroprusside (SNP) is a highly effective strategy in pulmonary and systemic hypertension crisis, both in adults and in neonates [1,2]. The most common SNP application is intravenous, but inhalation and endotracheal application is used as well [3,4]. The main mechanism of action may be explained by the release of NO which results in a dilation of small vessels both in the systemic and the pulmonary circulation. A well known side effect of NO is the inhibition of platelet function [5,6]. This interference of platelet activation is due to the stimulation of the soluble guanylate cyclase which increases the concentration of cGMP and inhibits the calcium-dependant platelet activation [7]. Just recently Jensen et al. published a study on the potential synergistic effect of Dipyridamole and NO donors, such as sodiumnitroprusside, to prolong the inhibition of thrombin-induced platelet shape change. [8] Similar results confirming the synergistic effect of NO donors and other antiplatelet drugs, could be demonstrated by Negrescu et al. [9]. In addition, Suzuki and coworkers suggest a synergistic effect of SNP and fibrinolytic agents [10]. In vitro studies have shown that NO inhibits platelet adhesion to endothelial cells [11] as well as platelet aggregation [12,13], platelet activation [5-7,14,15] and clot formation [16,17] and thrombus growth under shear stress [18]. However, these results could not always be confirmed by in vivo studies. Several studies have been performed investigating the mechanism of SNP action on platelet aggregation, but the exact means of interaction still is not understood [19,20]. Most authors confirm the NO dependent inhibition of platelet activation by SNP. Woods and coworkers clearly demonstrated that the platelet response to SNP in hypertensives is lower due to reduced sensitivity of the platelets to NO [21]. Sogo and coworkers showed that in the absence of endothelial cells the effect of SNP is exclusively cGMP dependent, which is in accordance with our results [22]. In addition this direct interference with platelet activity, cGMP stimulates cyclooxygenase activity in endothelial cells, resulting in increased concentrations of prostacyclins and the subsequent inhibition of platelet activation [23-25].

As well as this NO dependent effect other mechanisms seem to have an important impact on the inhibitory action of SNP in the presence of endothelial cells. Levin et al. already mentioned that the supernatant taken from SNP-incubated endothelial cells, had a surprising inhibitory effect on platelet aggregation not due to NO alone [26].

In the present study we wanted to evaluate the role of endothelial cells on the antiaggregatory effect of SNP. An additional aim was to investigate wether the time span between the incubation of platelets with SNP and measurement of the antiaggregatory effect provides an explanation for the sometimes contradictory results of in vitro and in vivo studies. The third goal was to test the reversibility of the antiaggregatory effects of SNP by cyanide and classical NO pathway inhibition.

## Material and methods

### Reagents

Sodium nitroprusside (SNP), Cyanide and ADP were purchased from Sigma laboratories and 1 H-(1,2,4)oxadia-zolo(4,3-a)quinoxalin-1-one (ODQ) was purchased from Alexis Biochemicals. PBS was obtained from Boehringer, Germany and Protaminhydrochloride (Protamin ICN 1000) was from ICN, Frankfurt, Germany. Acetyl sali-cylic acid (ASS) was obtained from Bayer, Leverkusen, Germany and indomethacin (Liomethacin) was purchased from Chiesi Pharmaceutica, Parma, Italy.

### Endothelial cell culture

For the endothelial cell experiments we used a well established configuration using human umbilical endothelial cells (HUVEC). HUVEC were isolated from fresh human umbilical cord vein by enzymatic disaggregation with collagenase, and were used between passages 2 and 5. HUVEC were cultured as previously described [27], the plates were washed with PBS before the blood sample was added and Heparin was neutralized with 75 I.U. of Protamin. Blood samples Venous blood was drawn from 4 healthy volunteers and coagulation was prevented immediately using 3.8% sodium citrate 1:10. The volunteers took no medication two weeks prior to blood sampling.

### Incubation with SNP

The effect of different concentrations of SNP and different incubation times was tested. After adding SNP to 5 mL of whole blood, samples were placed on culture plates. A new plate was used for each concentration and incubation time. Samples without SNP were used as controls. To study the effect of endothelial cells on aggregation, the incubation was performed on culture plates with, and without endothelial cells.

SNP concentrations were 100 μM, 500 μM and 1000 μM. Time course studies were performed with 100 μM SNP, and incubation times of the whole blood samples were 0, 1, 2 and 4 hours.

As total centrifugation time was 20 minutes (s. below) and SNP was added to the whole blood sample before centrifugation contact time between SNP and platelets was at least 20 minutes. Samples were kept at 37°C during the whole experiment.

### Mechanism of SNP – mediated inhibition of platelet function

We wished to study the mechanism by which SNP inhibits platelet aggregation. NO signal transduction by cGMP was inhibited using the guanylate cyclase inhibitor 1 H-(1,2,4) oxadiazolo(4,3-a)quinoxalin-1-one (ODQ). ODQ (100 μM) was added to the sample 2 minutes before adding 100 μM SNP to allow the reaction of ODQ with the guanylate cylcase. Platelet rich plasma was then obtained from the blood sample. The platelet function was tested using the Born method. Aggregation was induced with ADP (20 μM) after the platelet count had been adjusted to 250,000/μL.

To study whether SNP has an effect on the endothelial production of prostaglandins by activating cyclooxygenases (COX I and II), two cyclooxygenase inhibitors, acetylsalicylic acid and indomethacin were used. Endothelial cells were incubated for 5 min with 3 ml of either 1 mM acetylsalicylic acid or 10 μM indomethacin. Sufficient suppression of the cyclooxygenases in HUVEC by ASA or indomethacin has been previously described [28]. After thorough washing with PBS the experiments were repeated as described above.

To study the effect of cyanide on the effect of SNP, we added cyanide to whole blood in one set-up. In another experiment we added cyanide to platelet rich plasma (PRP) after the addition of ADP. A control group proved that cyanide itself has no effect on platelet aggregation.

### Platelet aggregation

For preparation of platelet-rich plasma the whole blood samples were centrifuged for 10 minutes at 800 r.p.m. Platelet-poor plasma was obtained by further centrifugation of PRP at 3800 r.p.m for 10 minutes. Platelet count was then adjusted with PPP to 250,000/μL. Platelet response to ADP (20 μM) was recorded with a Lumiaggregometer (Aggregometer 840, Elvi).

### Data analysis

Data represent the mean +/− SD of at least 4 experiments.

The levels of aggregation given in percent were detected every 20 seconds for 6 minutes. The following parameters were used for statistical analysis: For each experiment, all values of the aggregation curves were compared. Data were analyzed with Prism 3.0 using the Kruskal-Wallis-test and p ≤ 0.05 was accepted as being significant. In case of multiple comparisons p – values were corrected according to Dunn. Results were grouped according to incubation time: Group 1 = no incubation time, group 2 = incubation time of 1 hour, group 3 = incubation time of 2 hours, group 4 = incubation time of 4 hours.

## Results

The difference in platelet aggregation of controls with, and without, endothelial cells was not significant different. (Additional file 1 1^st^ and 3^rd^ column). We could show that ADP induced thrombocyte aggregation was intact even 4 h after blood sampling, with and without endothelial cells.

### Effect of SNP on platelet aggregation, effect of endothelial cells

After 1 hour of incubation SNP inhibited platelet aggregation in a dose-dependent manner. 100 μM, 500 μM and 1000 μM of SNP were tested (data not shown). This was statistically significant for all concentrations of SNP. These experiments were not repeated with endothelial cells. Time course studies were performed with 100 μM of SNP, as the 100 μmol curve at one hour was at the middle of normal aggregation and total inhibition, so changes in both directions would be detectable. A time dependent reduction of platelet aggregation could be detected with an inverse correlation to incubation time. Endothelial cells significantly increased the SNP – mediated inhibition of platelet function after 2 and 4 hours of incubation (Additional file 1 2^nd^ and 4^th^ column).

### Effect of ODQ on SNP mediated inhibition of platelet aggregation

Incubation with ODQ decreased the effect on platelet aggregation at 0 h of incubation time. ODQ did not inhibit the SNP mediated-effect on platelet aggregation later in the presence of endothelial cells. (Additional file 1 6^th^ column)

### Effect of COX-inhibition on SNP mediated reduction of platelet aggregation

Since endothelial cells play an important role in the SNP-induced inhibition of platelet aggregation, the effect of COX inhibitors and of ODQ was tested only after incubation with endothelial cells. The cox inhibitors acetyl salicylic acid and indomethacin (in the graph called COX I) did not influence the effect of SNP on platelet aggregation. The results were therefore combined. The pretreatment of endothelial cells with COX inhibitors did not interfere with the effect of SNP incubation (Additional file 1 7^th^ column).

### Effect of cyanide on SNP mediated inhibition of platelet aggregation

Cyanide (2000 μM) alone did not influence platelet aggregation. Cyanide added to whole blood prior to addition of SNP repressed the effect of SNP in all cases. The addition of cyanide after the preparation of platelet-rich plasma, prior to the addition of ADP only inhibited the SNP effect of the endothelial cell experiments (Additional file 1 5^th^ column).

## Discussion

In our study we could clearly demonstrate that endothelial cells play an important role in the antiaggregatory effect of SNP. Incubation with endothelial cells increased both the inhibitory effect on platelet aggregation and the duration of this effect. Levin et al. already mentioned that the supernates taken from SNP-incubated endothelial cells had an inhibiting effect on platelet aggregation [26]. They speculated that their data could be explained by the synergy of NO and PGI2 [26]. The Levin study used supernatants of SNP-incubated endothelial cells and did not preincubate the endothelial cells with cyclooxygenase inhibitors, which may have led to different results. In contrast to their experimental setting, the platelets in our study were in direct contact with the endothelial cells during SNP incubation, which suggests possible additional interactions between platelets and endothelial cells. It has been well established that PGs of the E series and PGI2 inhibit platelet aggregation by counteracting the proaggregatory effect of thromboxane A2 [29]. This could explain the additional inhibitory affect of the endothelial cells in the presence of NO. However, this explanation seems insufficient, as the preincubation of endothelial cells with cyclooxygenase inhibitors, suppressing the PGI2 production, did not change the SNP effect as expected. In an earlier study we could show that the effect of other NO-donors, such as SNAP, is increased by the PG pathway. Induction of PGI2 via NO, following reduced platelet aggregation, could be blocked by preincubation of the endothelial cells with cyclooxygenases [28]. The effect of SNP was not attenuated by the preincubation of the endothelial cells with a cyclooxygenase inhibitor. This is in contrast to the conclusions of Levin and coworkers [26]. Sogo et al. could clearly demonstrate that, in the absence of endothelial cells, the effect of SNP is exclusively cGMP-dependent [22], which should be the case if the effect is due to NO release. In our setting the effect of SNP is not attenuated by the guanylate cyclase inhibitor ODQ, which is supposed to block the cGMP-dependent NO effect. This opposes the hypothesis that the effect of SNP is exclusively cGMP dependent in the presence of endothelial cells. These results highlight the importance of the endothelial cell in SNP metabolism and platelet interaction. Our data do not allow us to decide which pathway is responsible for the results measured. Because the effect of endothelial cells on platelet aggregation could not be reversed by incubation with a COX-inhibitor, we speculate that activation of endothelial cyclooxygenases by SNP may not be too important for the antiaggregatory effect of SNP.

Our results without endothelial cells show an effect of SNP on platelets which is in accordance with other in vitro studies [7,26,30].

Another interesting fact is that the addition of cyanide is able to reverse the effect, not only after immediate administration, as shown by Brune et al. [5], but even after 4 h of incubation with SNP. This effect has not been described before in the literature. Although we do not have an explanation for this observation, we speculate that a cGMP independent pathway may be responsible for this effect.

In summary, our results show that endothelial cells play an important role in the SNP- mediated inhibition of platelet activation, which can be reversed only by cyanide. We could demonstrate that this effect is not mediated by activation of endothelial cyclooxygenases, as the inhibition of cyclooxygenases had no influence on the results. It is not dependent on cGMP, as it is not influenced by the guanylate cyclase inhibitor ODQ. Thus we suggest a cGMP independent, possibly not yet known pathway for platelet inhibition by SNP/endothelial cells.

The identification of this unknown antiaggregatory metabolite could be of great interest, since its effect on ADP-dependent platelet aggregation is long-lasting, but reversible by cyanide. We speculate that other less toxic ion metabolites could also reverse this effect, thus offering the opportunity for antidot treatment.

## Competing interests

All authors have read the manuscript and conflict of interest statement and have approved their submission for publication; all authors declare that they do not have competing interests.

## Authors’ contributions

H-GT was performing the experiments and wrote the manuscript. MR and JK established the born method for measuring platelet aggregation and gave helpful input on pre-analytical pitfalls when working with platelets. WR and JD established the endothelial cultures in the lab and gave relevant input on the test up during incubation. All authors have read and approved the final manuscript.

## Supplementary Material

Additional file 1 Comparison of aggregation at timepoints 0, 1, 2 and 4 hours. Click here for file
