# Supplementary material for: Endothelial cells influence the sodium nitroprusside mediated inhibition of platelet aggregation by an as yet unkown pathway
Source: Thromb J. 2012 May 7;10:6. doi: 10.1186/1477-9560-10-6 (PMC3528661; doi:10.1186/1477-9560-10-6)
Supplement: Additional file 1 — Comparison of aggregation at timepoints 0, 1, 2 and 4 hours. [file 1477-9560-10-6-S1.pdf]

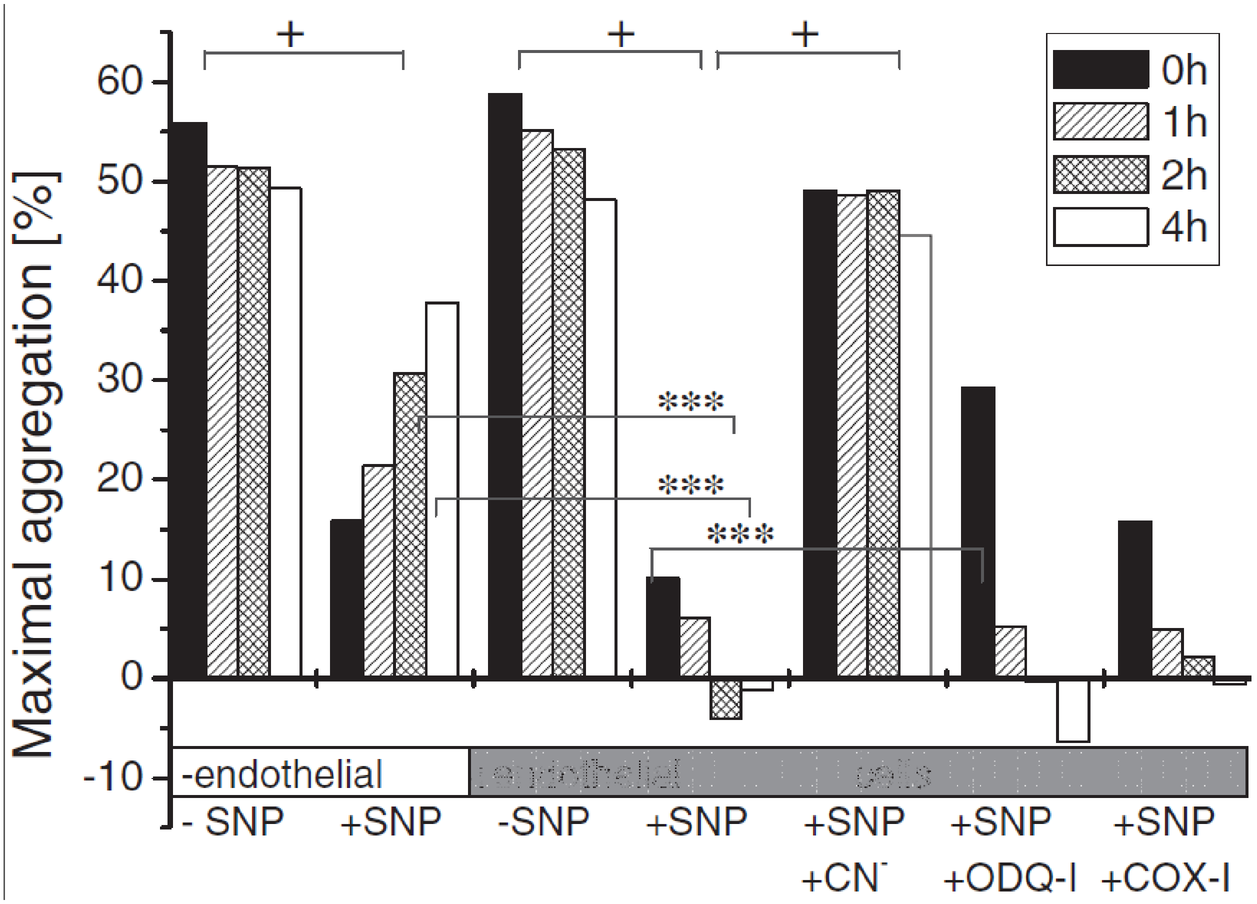

Additional file 1 Comparison of aggregation at timepoints 0, 1, 2 and 4 hours in each column With (+) or withhout (-) endothelial cells and with (+) or without (-) additional substances + comparing columns in total, or *** at single time points *** $p<0.001$, + $p<0.01$ at all time points
